# Antiepileptic drugs in glioblastoma survival: dichotomic or treatment and mechanism of action-dependent variable?

**DOI:** 10.1093/noajnl/vdag035

**Published:** 2026-02-11

**Authors:** Oktay Genel, Sultan Alzarouni, Asfand Baig Mirza, Prajwal Ghimire, Sarah Murden, Yasir A Chowdhury, Ali Elhag, Katia Cikurel, Dorothy Joe, Gerald Finnerty, Vishal Manik, Angela Swampillai, Omar Al-Salihi, Kazumi Chia, Lucy Brazil, Samantha Forner, Jennifer Glendenning, Richard Gullan, Keyoumars Ashkan, Ranjeev Bhangoo, Francesco Vergani, José Pedro Lavrador

**Affiliations:** Department of Neurosurgery, King’s College Hospital Foundation Trust, London, UK; Cambridge University Hospitals Foundation Trust, Cambridge, UK; Department of Neurosurgery, King’s College Hospital Foundation Trust, London, UK; Department of Neurosurgery, King’s College Hospital Foundation Trust, London, UK; Department of Neurosurgery, King’s College Hospital Foundation Trust, London, UK; Department of Neurology, King’s College Hospital Foundation Trust, London, UK; Department of Basic and Clinical Neuroscience, Institute of Psychiatry, Psychology, Neuroscience at King’s College London and Department of Neurooncology, Guy’s Cancer Centre, London, UK; Department of Basic and Clinical Neuroscience, Institute of Psychiatry, Psychology, Neuroscience at King’s College London and Department of Neurooncology, Guy’s Cancer Centre, London, UK; Department of Basic and Clinical Neuroscience, Institute of Psychiatry, Psychology, Neuroscience at King’s College London and Department of Neurooncology, Guy’s Cancer Centre, London, UK; Department of Basic and Clinical Neuroscience, Institute of Psychiatry, Psychology, Neuroscience at King’s College London and Department of Neurooncology, Guy’s Cancer Centre, London, UK; Department of Neurooncology, Kent Cancer Centre, UK; Department of Neurooncology, Kent Cancer Centre, UK; Department of Neurosurgery, King’s College Hospital Foundation Trust, London, UK; Department of Neurosurgery, King’s College Hospital Foundation Trust, London, UK; Department of Neurosurgery, King’s College Hospital Foundation Trust, London, UK; Department of Neurosurgery, King’s College Hospital Foundation Trust, London, UK; Department of Neurosurgery, King’s College Hospital Foundation Trust, London, UK

**Keywords:** antiepileptic drugs, glioblastoma, overall survival

## Abstract

**Background:**

Recent evidence has demonstrated a tight relationship between neuronal activity and glioblastoma (GBM) growth, involving novel mechanisms such as neuron-glioma synapses and tumor microtube networks. Seizure activity and antiepileptic drug (AED) usage are highly prevalent among GBM patients. In this study, we investigate the impact of AEDs and their mechanism of action on overall survival (OS) in a cohort of patients treated for GBM.

**Methods:**

We performed a retrospective, single-center study of a cohort of histopathologically proven GBM patients at a tertiary center. Multivariate analyses were performed at 4 different timepoints by (1) patients who did and did not use AEDs, (2) use of individual AEDs, and (3) use of AEDs with the same mechanism of action.

**Results:**

A total of 236 patients were included in the analysis, 178 of which were on anti-epileptics (75.4%). There was no significant impact of AEDs overall in OS—median survival was 16.2 months for patients taking AEDs and 13.8 months for patients not. Being on a voltage-gated sodium channel (VGNC) blocker seemed to confer a significant survival advantage at 24 months when compared with patients not on AEDs (hazard ratio [HR] = 0.67, *P* = .045). This significance was, however, lost when corrected for covariates (multivariable HR = 1.01, confidence interval [CI] = 0.67-1.54, *P* = .953). While patients on adjuvant treatment had a higher OS, this was only the case in patients ­taking AEDs.

**Conclusion:**

There was no significant effect of AEDs on OS, VGNC blockers trended toward significance at 24 months. However, a link may exist between AEDs and the impact of adjuvant treatments.

Key Points

- There was no significant impact of AEDs as a whole in GBM overall survival.
- Patients on voltage-gated sodium channel blockers trended toward higher survival at 24 months.
- AEDs may have a link with the effectiveness of adjuvant treatments.

Importance of the Study

Multiple high-impact studies have recently demonstrated a critical relationship between neuronal activity and GBM growth. Many patients experience seizures during their treatment, and an even greater number of them receive AEDs. The impact of AEDs on survival of GBM patients is however poorly reported in the literature. This single-center retrospective study of 236 GBM patients reports that there is no significant impact of AEDs on survival. However, a subgroup of patients (those on VGNC blockers) had higher OS at 24 months. Most importantly, in our study, AEDs seem to modulate the impact of adjuvant treatments. This result opens the way for further studies investigating novel approaches to enhance the effectiveness of adjuvant treatment effectiveness, namely, by the incorporation of AEDs in the treatment regimen of GBM patients.

Seizures are common in glioblastoma (GBM) patients. A quarter of patients experience seizures in the preoperative period,^1^ and about 60% to 70% of patients experience seizures at some point during their treatment.^2^^,^^3^ Recent clinical studies have explored the relationship between neuronal activity and GBM growth.^4^ Initial evidence coming from a mouse model demonstrates that optogenetically modulated neuronal activity drives glioma growth, via secretion of mitogens such as Neuroligin-3.^5^ Subsequent evidence shows the existence of synapses between neuronal axons and glioma cells,^6^ and reveals that those synapses drive tumor progression.^7^ Furthermore, glioma cells are integrated in a physical network via tumor microtubes enabling chemical communication between tumor cells and resistance to current therapies.^8^ From a translational perspective, increased neuronal excitability was reported in glioma-infiltrated brains^6^ and patients who have tumors that are highly integrated into cerebral neuronal circuits have worse prognosis.^9^ This recent paradigm shift brought about by our understanding that glioma growth is driven by neuronal activity opens the path for investigating the therapeutic role of antiepileptic drugs (AEDs) in GBM.

Antiepileptic drugs have a wide range of pharmacological action mechanisms which may have implications in GBM (Figure 1). Several clinical studies have investigated survival in patients taking AEDs.^14^ Some AEDs—such as sodium valproate—were reported to improve survival while other AEDs like levetiracetam or lamotrigine showed mixed results.^14^^,^^15^ While the EANO and SNO currently advise against the use of AEDs prophylactically,^16^ the current clinical evidence is conflicting and failed to strongly support or refute the therapeutic use of AEDs in GBM. In this present study, we investigate the impact of AEDs and their mechanism of action on survival in a cohort of patients treated for GBM.

**Figure 1.**
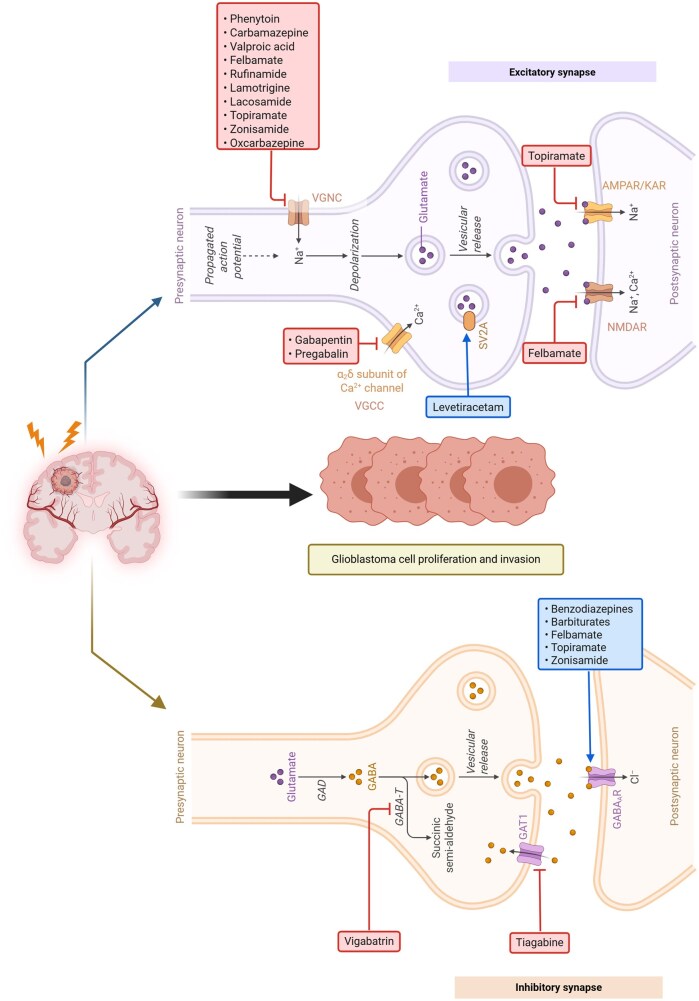
AEDs in glioblastoma. The illustration describes AEDs with their site of action in excitatory and inhibitory synapses in patients with glioblastoma. (VGNC: voltage gated sodium channel; VGCC: voltage gated calcium channel; SV2A: synaptic vesicle glycoprotein 2A; GABA-T: 4-aminobutyrate transaminase; GABA: gamma-aminobutyric acid; NMDAR: N-methyl-D-aspartate receptor; AMPAR: α-amino-3-hydroxy-5-methyl-4-isoxazolepropionic acid receptor; KAR: killer activation receptor) (created in Biorender.com). Levetiracetam is known to inhibit SV2A.^10^ Sodium valproate enhances GABA activity and blocks voltage-gated sodium channels.^11^ Carbamazepine, lamotrigine and phenytoin are voltage-gated sodium channel blockers.^12^ Pregabalin and gabapentin only have a modest effect on GABA activity, and instead exert their antiepileptic properties through the inhibition of VGCC containing the α2δ1 subunit.^13^ Abbreviation: AED, antiepileptic drug.

## Methods

### Patient Selection and Data Collection

We performed a retrospective tertiary single-center study of a cohort of adult GBM patients diagnosed according to the latest WHO Classification between 2014 and 2019.^17^ All patients were IDH-wildtype. Patients who did not have an IDH mutation status were excluded alongside patients who were IDH-mutant. No patients with recurrent GBM were included in the study. Thirty-four of the included patients had 1 or more re-do surgeries. The median age of those patients was 60, while the median OS was 14.9 months. No patients treated since 2019 were included to account for the potential bias of long survivors.^18^ In our study, long survivors were defined as patients having an OS greater than 5 years.^19^

For each patient, data were collected for age, gender, date of diagnosis, and date of death (if applicable). Data were collected by a single author on the use of antiepileptics at any point, for any duration (levetiracetam, sodium valproate, carbamazepine, lamotrigine, phenytoin, gabapentin, and pregabalin), and indication for use. Patients who took an antiepileptic at any point were included, and the duration of use in months was estimated by subtracting the date of first prescription on the patient records from the date of the last clinical encounter or prescription confirming AED use. Overall survival (OS) was defined as the difference in months between date of diagnosis and death or last hospital interaction. Extent of resection (EOR) was calculated and patients were classified into subgroups according to the Response Assessment in Neuro-Oncology (RANO) criteria for EOR.^20^ Patients were grouped by antiepileptic groups as follows: SV2A modulator (levetiracetam), voltage-gated sodium-channel blockers (VGNC—carbamazepine, lamotrigine, phenytoin), voltage-gated calcium channel blockers (VGCC—gabapentin and pregabalin), and antiepileptics both increasing GABA and blocking sodium channels (sodium valproate in our study).^21^ Also, we have analyzed the impact of the AED medication in the different types of adjuvant treatment after surgical resection (chemoradiotherapy—CRT; chemotherapy—CTx; Radiotherapy—RTx; and no adjuvant treatment).

### Statistical Analysis

Survival analyses were performed using the open-source software JASP1 and Jamovi2-7. JASP was used to generate Kaplan-Meier and Log-rank tests. Jamovi was used to generate multivariate analyses using the Cox proportional hazards model. R studio and the survival package were used to calculate 6-month, 12-month, 18-month, and 24-month survival. Missing values were handled using a multiple imputation method with the mice R package. Multivariate analyses were performed at 4 different timepoints by (1) patients who did and did not use AEDs, (2) use of individual AEDs, and (3) use of AEDs with the same mechanism of action. Data were corrected for the following covariates due to their clinical significance on GBM survival: age, gender, type of resection, preoperative and postoperative performance status, RANO criteria, and methylated-DNA-protein-cysteine methyltransferase (MGMT) status.

## Results

### Demographics

A total of 236 patients with histopathologically confirmed GBM were included in the analysis (Table 1). The mean age of included patients was 56.4 years old (133 male and 103 female patients, SD = 12.7). The median survival for all patients was 15.2 months (mean = 22.06, SD = 23.03). The RANO classification grade for EOR was 3B in 60 patients (25.4%), 3A in 38 patients (16.1%)—submaximal contrast enhancement resection, 2A in 29 patients (12.3%), and 2B in 8 patients (3.4%)—maximal contrast enhancement resection. The type of resection was total resection in 98 patients (41.5%), subtotal resection in 136 patients (57.6%), biopsy in 1 patient (0.4%), and unknown in 1 patient (0.4%). Most tumors were located in the temporal (35.2%) or frontal lobe (25.3%). The preoperative WHO performance status was predominantly 0 and 1 (48.3% and 36.4%, respectively), whereas the postoperative performance status was predominantly 1 (40.7%), followed by 0 (25.6%). The proportion of methylated (47%) and unmethylated (48.7%) tumors was similar. Most patients received adjuvant chemoradiotherapy (58.5%), 25% of patients received no adjuvant treatment, 8.9% of patients received chemotherapy, only and 8% of patients received radiotherapy only. Among the 58 patients who did not have any adjuvant treatment, 20.6% had a preoperative performance score of 2 or above, whereas it is only 12.7% in the entire cohort.

**Table 1.** Demographic and clinical characteristics of patients included in the study, for all patients, patients on AEDs, patients not on AEDs, and patients on VGNC blockers.

Variables	Total		AED		No AED		VGNC	
	*N*	%	*N*	%	*N*	%	*N*	%
**Gender**								
Female	103	43.6	78	43.8	25	43.1	20	41.7
Male	133	56.4	100	56.2	33	56,9	28	58.3
**Location**								
Frontal	62	26.3	51	28.7	11	19.0	11	22.9
Temporal	83	35.2	63	35.4	20	34.5	23	47.9
Parietal	39	16.5	24	13.5	15	25.9	8	16.7
Cerebellum	2	0.85	2	1.1	0	0	1	2.1
Occipital	12	5.08	9	5.1	3	5.2	2	4.2
Intraventricular	1	0.42	1	0.6	0	0	0	0
Multiple	34	14.4	25	14.0	9	15.5	3	6.3
**Preoperative WHO performance score**								
0	114	48.3	86	48.3	28	48.3	29	60.4
1	86	36.4	65	36.5	21	36.2	14	29.2
2	26	11.0	18	10.1	8	13.8	3	6.3
3	4	1.69	4	2.2	0	0	0	0
4	0	0.00	0	0	0	0	0	0
5	0	0.00	0	0	0	0	0	0
**Postoperative WHO performance score**								
0	61	25.9	50	28.1	11	19.0	8	16.7
1	96	40.7	67	37.6	29	50.0	19	39.6
2	23	9.75	19	10.7	4	6.9	4	8.3
3	24	10.2	18	10.1	6	10.3	9	18.8
4	7	2.97	7	3.9	0	0	1	2.1
5	11	4.66	9	5.1	2	3.4	4	8.3
**RANO (2023)**								
2A	29	12.3	23	12.9	5	8.6	7	14.6
2B	8	3.39	6	3.4	2	3.4	3	6.3
3A	38	16.1	25	14.0	11	19.0	6	12.5
3B	60	25.4	44	24.7	13	22.4	14	29.2
**Type of resection**								
Total	98	41.5	62	34.8	26	44.8	16	33.3
Subtotal	136	57.6	114	64.0	32	55.2	32	66.7
Biopsy	1	0.4	1	0.6	0	0	0	0
Unknown	1	0.4	1	0.6	0	0	0	0
**MGMT methylation status**								
Unmethylated	115	48.7	81	45.5	34	58.6	17	35.4
Borderline	3	1.3	2	1.1	1	1.7	1	2.1
Methylated	111	47.0	88	49.4	23	39.7	27	56.3
Missing	7	3	7	3.9	0	0	3	6.3
**Adjuvant treatment**								
None	58	25	39	21.9	19	32.8	6	12.5
Chemotherapy	21	8.89	14	7.9	5	8.6	3	6.3
Radiotherapy	19	8.05	15	8.4	6	10.3	4	8.3
Chemotherapy & Radiotherapy	138	58.5	110	61.8	28	48.3	35	72.9
**AED**								
AED use	178	75.4	178	100	-	-	48	100
No AED use	58	24.6	0	0	-	-	0	0
**AED number**								
1	122	68.5	122	68.5	-	-	11	22.9
2	47	15.8	47	15.8	-	-	29	60.4
3	5	2.81	5	2.81	-	-	4	8.3
4	4	2.25	4	2.25	-	-	5	10.4
**AED type**								
Keppra/Levetiracetam	156	66.1	156	66.1	-	-	32	66.7
Phenytoin	22	9.32	22	9.32	-	-	22	45.8
Lamotrigine	19	8.05	19	8.05	-	-	19	39.6
Sodium valproate	15	6.36	15	6.36	-	-	5	10.4
Carbemazapine	13	5.51	13	5.51	-	-	13	27.1
Pregabalin	12	5.08	12	5.08	-	-	2	4.2
Gabapentin	8	3.39	8	3.39	-	-	1	2.1
Phenobarbital	1	0.42	1	0.42	-	-	1	2.1
Topiramate	1	0.42	1	0.42	-	-	1	2.1
**AED mechanism**								
SV2A modulator	156	66.1	156	66.1	-	-	32	66.7
Voltage-gated Na channel blocker	48	20.3	48	20.3	-	-	48	100
Voltage-gated Ca channel blocker	18	7.63	18	7.63	-	-	3	6.3
Increase GABA and VG-Na channel blocker	16	6.78	16	6.78	-	-	6	12.5
Increase GABA	1	0.42	1	0.42	-	-	1	2.1
**Indication for AED medication**								
Seizure-related reason	103	57.6	103	57.6	-	-	38	79.2
Prophylactic AED use	53	30.0	53	30.0	-	-	6	12.5
Unknown	16	9.0	16	9.0	-	-	3	6.3
Other causes	6	3.4	6	3.4	-	-	1	2.1

Abbreviations: AED, antiepileptic drug; MGMT, methylated-DNA-protein-cysteine methyltransferase; RANO, Response Assessment in Neuro-Oncology; VGNC, voltage-gated sodium channel.

The mean age of included patients was 56.4 years old (133 male and 103 female patients, SD 12.7). The median survival for all patients was 15.2 months (mean = 22.06, SD = 23.03). Data for the preoperative performance score were unavailable in 6 patients, and the postoperative performance score was unavailable in 14 patients.

A total of 178 patients (75.4%) were on antiepileptics—129 patients (72.5%) on monotherapy and 58 patients (30.7%) on polytherapy: 156 were on levetiracetam (66%), 22 on phenytoin (9.3%), 19 on lamotrigine (8.1%), 15 on sodium valproate (6.3%), 13 on carbamazepine (5.5%), 12 on pregabalin (5.1%), and 8 on gabapentin (3.3%). The indication for starting AEDs was seizure-related treatment 103 patients (57.6%)—generalized tonic-clonic seizure in 52 patients (29%), other seizures in 51 patients (28.6%)—prophylaxis in 53 patients (30%), unknown in 16 patients (9%), and other causes (headache, trigeminal neuralgia, confusion, and neck pain) in 6 patients (3.4%).

### Impact of AEDs on OS

**Figure 2.**
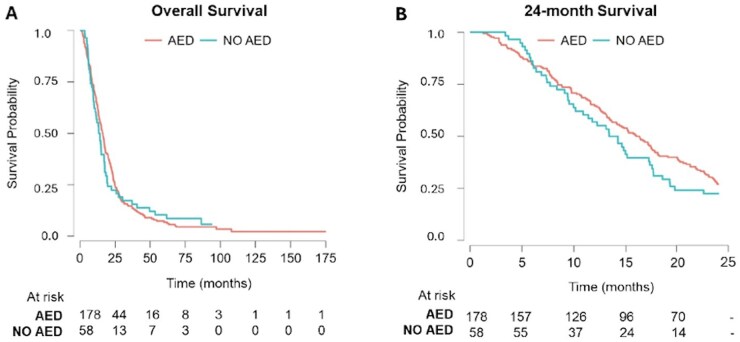
Kaplan-Meier curves comparing survival of glioblastoma patients taking AEDs versus not taking AEDs. (**A**) Kaplan-Meier curve for overall survival in months. Median survival was not significantly different between patients taking AEDs and patients not taking AEDs (16.17 months vs 13.82 months, respectively, hazard ratio = 1.04 (95% confidence interval = 0.77-1.42, *P* = .78). (**B**) Kaplan-Meier curve for 24-month survival. Patients taking AED shown in orange, NO AED in blue. Number of patients at risk for both groups are shown below the Kaplan-Meier curve. There was no difference in survival at different time points. Survival at 6 months was 86.2% in patients not taking AEDs and 84.8% in patients taking AEDs (*P* = .66). At 12 months, survival was 56.9% in patients not taking AEDs and 65.2% in patients taking AEDs (*P* = .32); at 18 months, 36.2% in patients not taking AEDs and 43.8% in patients taking AEDs (*P* = .16); at 24 months, 22.4% in patients not taking AEDs and 27.0% in patients taking AEDs (*P* = .27). Abbreviation: AED, antiepileptic drug.

Overall, our results show that there is no significant impact of AEDs as a whole in GBM OS—median survival was 16.17 months for patients taking AEDs and 13.82 months for patients not taking AEDs with a hazard ratio (HR) of 1.04 (95% confidence interval [CI] = 0.77-1.42, *P* = .78). (Figure S1 and Figure 2). No statistical significance was found when analyzing survival at different time periods. Survival at 6 months was 86.2% in patients not taking AEDs and 84.8% in patients taking AEDs (*P* = .66). At 12 months, survival was 56.9% in patients not taking AEDs and 65.2% in patients taking AEDs (*P* = .32); at 18 months, 36.2% in patients not taking AEDs and 43.8% in patients taking AEDs (*P* = .16); at 24 months, 22.4% in patients not taking AEDs and 27.0% in patients taking AEDs (*P* = .27).Also, the indication for AEDs medication had no significant impact in survival. Patients using AEDs for seizure treatment had a median survival of 15.20 months compared with 16.83 months for prophylaxis (*P* = .516). Analysis of patients taking AEDs for prophylaxis/nonseizure treatment (usually lower dosage) versus seizure treatment (usually higher dosage) showed no significant impact on survival (*P* = .886), with a median survival of 17.18 and 13.88 months, respectively (Figure S8).

**Figure 3.**
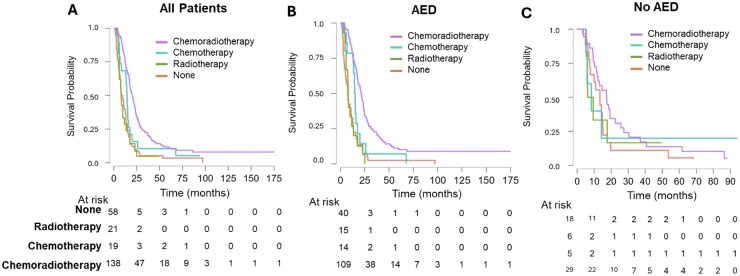
Kaplan-Meier curves comparing overall survival in months for different adjuvant treatments for all patients, taking and not taking AEDs. (**A**) For the entire cohort, median survival was statistically longer in patients having chemoradiotherapy (20.2 months) when compared with patients who had chemotherapy only (14.4 months), radiotherapy only (8 months), or no adjuvant treatment (9.02 months). (**B**) AED group: patients who underwent adjuvant chemoradiotherapy had statistically significant longer median survival when compared with other adjuvant treatments: OS—chemoradiotherapy: 21.2 months vs chemotherapy—14.9 months vs radiotherapy 8 months vs no adjuvant treatment: 7.5 months, *P* < .001. (**C**) No AEDs group: there was no difference in the overall survival with different treatments: OS—CRT: 17.3 months vs CTx-only: 8.4 months vs RT-only: 7.8 months vs no adjuvant treatment: 13.4 months, *P* = .585. Abbreviations: AED, antiepileptic drug; OS, overall survival.

**Figure 4.**
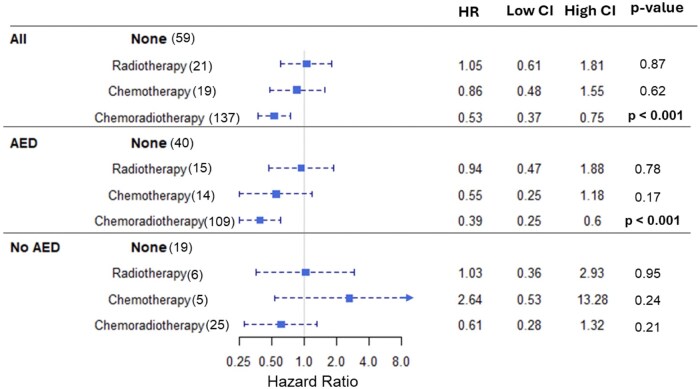
Forest plot of a multivariate COX regression test per adjuvant treatment corrected for covariates known to be clinically relevant: age, gender, type of resection, preoperative and postoperative performance status, Response Assessment in Neuro-Oncology (RANO), and methylated-DNA-protein-cysteine methyltransferase (MGMT) status. Model used: h(t)=h0(t) exp(β1X1+β2X2+⋯+βkXk). Abbreviations: AED, antiepileptic drug; CI, confidence interval.

For the whole cohort, median survival was statistically longer in patients having chemoradiotherapy (20.2 months) when compared with patients who had chemotherapy only (14.4 months), radiotherapy only (8 months), or no adjuvant treatment (9.02 months). The performance score did not significantly influence treatment choice; around 30% of patients who did not receive any adjuvant treatment had a high performance status, while 40% of patients who received adjuvant treatment had a high performance status. A subgroup analysis according to the AED medication revealed that in the no-AEDs patient group, different types of adjuvant treatment were not associated with significant differences in the OS: OS—CRT: 17.3 months versus CTx-only: 8.4 months versus RT-only: 7.8 months versus no adjuvant treatment: 13.4 months, *P* = .585. In the AED group, patients who underwent adjuvant chemoradiotherapy had a statistically significant longer median survival when compared with other adjuvant treatment modalities: OS—CRT: 21.2 months versus CTx—14.9 months versus RT 8 months versus no adjuvant treatment: 7.5 months, *P* < .001) (Figures 3 and 4, Figures S2 and S3). The covariate forest plot (Figure S6) shows that established prognostic factors behave as expected: Better preoperative and postoperative KPS, more extensive resection (favorable RANO/EOR), MGMT methylation, and younger age are associated with improved survival, and these effects are similar in the overall cohort and when stratified by AED use. Performing an analysis excluding patients who did not undergo adjuvant treatment (Figure S7) did not show statistically significant differences (median survival was 18.10 months with AEDs vs 14.43 months for no AEDs, HR = 1.05; 95% CI = 0.68-1.62, *P* = .81) compared with all patients (median survival; AED: 16.17 months vs no AEDs: 13.82, HR = 1.04; 95% CI = 0.77-1.42, *P* = .78).

### AED Mechanism of Action and OS

**Figure 5.**
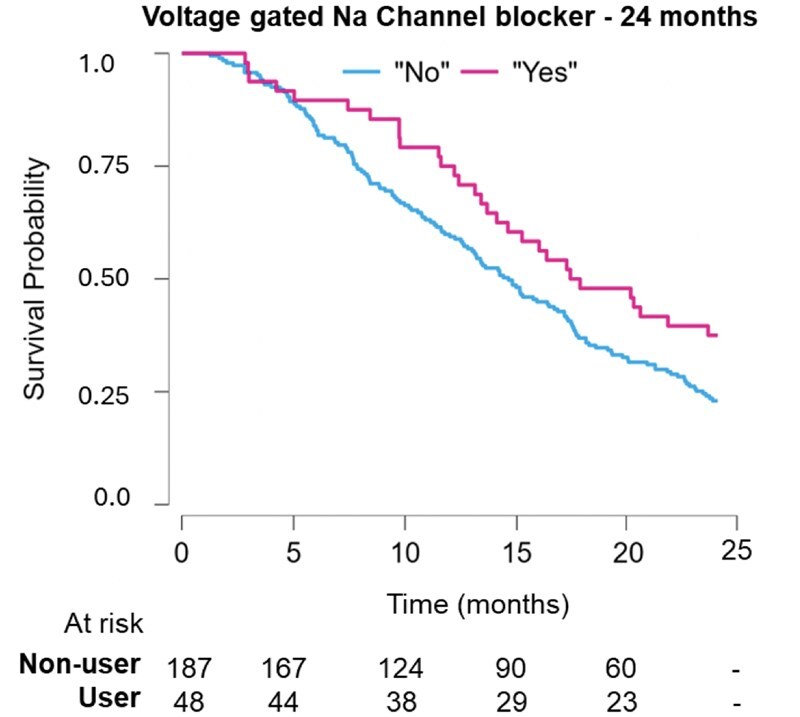
Kaplan-Meier plot for Voltage gated Na channel blocker.

We then investigated the impact of AEDs on OS based on specific groups of antiepileptics (Figures S4 and S5). The most representative mechanism of action among the prescribed AEDs (SV2 Modulation, 66.1% of patients on AEDs) had no significant impact in OS (*P* > .05). It was observed that VGNC did not hold proportionality, meaning that the HR changed over time (Schoenfeld individual test, *P* = .012); this was true for the period after 24 months (Schoenfeld individual test before 24 months, *P* = .52); hence, a cumulative landmark analysis was performed for up to 24 months where proportionality was encountered. All other mechanism of action did not change with time (Schoenfeld individual test, *P* > .05). The survival at 24 months was significantly higher in the VGNC blocker group (20.3% of patients on AEDs) when compared with patients not on AEDs (HR = 0.67, 33% risk of death reduction, 95% CI = 0.45-0.99, *P* = .045) (Figure 5). When corrected for covariates, this significance was lost (multivariable HR = 1.01, CI = 0.67-1.54, *P* = .953). In the same group, there is also a trend toward a significantly positive result at 12 months (HR = 0.56, CI = 0.31-1.03, *P* = .064).

## Discussion

The implications of AEDs in GBM survival can be more complex than an overarching effect of this type of medication and potentially go behind a single-agent effect. Those results seem to point to a potential link between AEDs and the impact of adjuvant treatment in OS. The AEDs significantly improve the OS in patients in the CRT group in comparison with the other treatment groups. This effect tends to be more pronounced with specific mechanisms of action: VGNC use trended toward longer OS at 24 months after diagnosis when compared with patients on no-AEDs.

In this study, OS is not influenced by AED intake. Despite growing interest, there is a lack of clinical trials to strongly support or refute the therapeutic use of AEDs. Large studies support the absence of changes in survival with AEDs such as sodium valproate, levetiracetam, or lamotrigine.^22^ Rigamonti et al have investigated the survival difference between patients taking enzyme-inducing and non-enzyme-inducing AEDs.^23^ While we have instead grouped patients by mechanism of AED, both studies support the overall lack of influence of AEDs on survival. Increased survival of patients on AEDs, especially sodium valproate, is reported in some studies.^24^^,^^25^ Our study refutes this association and is supported by a large analysis of prospective clinical trials.^26^

The lack of effect of AEDs in OS of GBM patients could have several explanations. First, AEDs have multiple mechanisms of action which exert diverse effects on tumor neurobiology. Those effects are often synergistic,^27^ adding to the significant amount of mechanistic overlap and incertitude regarding those complex downstream mechanisms.^28^ In addition, one could speculate that while AEDs influence GBM biology, they do not target the key mechanisms that make tumors resistant to treatment. Indeed, GBM stem cells (GSCs) are known to initiate tumors^29^ and to promote resistance to radiotherapy and ­chemotherapy.^30^ Quiescent GSCs in particular are known to drive recurrence as they escape temozolomide by being arrested in the G_0_ phase of the cell cycle,^31^ whereas temozolomide arrests proliferating cells at the G_2_-M phase boundary.^32^ One study describes no antiproliferative effect of one of the AEDs mentioned in our study, carbamazepine.^33^ We therefore attempted to add granularity to our analyses by organizing AEDs according to interactions with adjuvant oncological treatment and mechanisms of action.

The potential modulatory effect of AEDs in the effect of CRT *versus* the other types of adjuvant treatment is relevant not only because of the significance in this subgroup but particularly by its validation as per the absence of significance in the no-AEDs group. This interaction has been a topic of basic and translational research. Levetiracetam has been shown to potentiate the effect of temozolomide through inhibition of histone deacetylases,^34^^,^^35^ which are known to promote cancer development.^36^ Similar effects were reported with valproic acid^37^^,^^38^ and perampanel.^39^ Valproic acid has also been shown to radiosensitize tumors and improve survival.^40^ If we consider the lack of impact of AEDs on OS in the univariate dichotomic analysis, these results suggest the potential benefit lies in the interaction between the AEDs and the adjuvant treatments (in particular the CRT) and not in AEDs as a whole on their own.

With regard to mechanism of action, our study shows that VGNC blockers improve survival at 24 months of follow-up (Figure S4). Forty percent of patients taking VGNC blockers were alive at 24 months, compared with 25% of patients not taking VGNC blockers. In the GBM literature, sodium appears to be an electrolyte closely associated with survival. Numerous studies have shown that inhibition of sodium ion channels is associated with reduced aggressiveness of GBM in vitro.^41^ One study reports shorter survival in patients with mutations in the sodium channel genes. It is however limited by the low sample size and the lack of statistical significance when adjusted for IDH1 status.^42^ This central effect of sodium may stem from the fact that, similar to healthy brain, GBM is an electrically active tissue, which needs this activity to invade the brain.^43^ Depleting the GBM from its invasiveness via inhibition of sodium and calcium channels is therefore an appealing option. Calcium is indeed another ion heavily involved in GBM progression,^44^ and targeting it has shown preclinical promise.^45^ Nevertheless, in our cohort, patients treated with VGCC blockers did not have better survival than patients not on VGCC blockers. The low representation of this AED mechanism of action in our cohort (*n* = 18, 7.6% of the study population) is a potential factor that precludes further considerations about this group in this study.

The findings in our study have several important limitations. First, the retrospective nature of our study and its small sample size limited the power of our analyses, and we were not able to specifically assess each individual drug or group of drugs per adjuvant treatment group. Our study was also limited by the lack of data about dose and duration of each AED, owing to patients having follow-up in local institutions. Therefore, we could not identify and exclude patients who received AEDs for a short period of time in the emergency setting, or patients in whom AEDs were discontinued due to toxicity. Furthermore, patients see their dosage change frequently during treatment, which makes each individual difficult to compare with the rest of the cohort. We acknowledge the importance of various other molecular characteristics in GBM survival (such as TERT and EGFR alterations) but unfortunately could not report analyses due to a high amount of missing or unavailable data. Our analyses were not controlled for second line treatment (after recurrence), for timing relative to diagnosis or surgery, or for seizure frequency, which is independently associated with morbidity and mortality in GBM.^46^ Nevertheless, we provide a single-center, same-MDT cohort of a substantial number of GBM patients.

In summary, our study reports no significant difference in survival for individual AEDs and individual groups of AEDs. While drawing meaningful conclusions at this stage may not be sensible, our study raises important hypotheses regarding a potential role of AEDs in the survival of GBM patients, warranting further, higher-power analyses.

## Supplementary Material

vdag035_Supplementary_Data

## Data Availability

Anonymized data may be available upon reasonable request. Please email Dr Oktay Genel at oktay.genel@nhs.net.
