# Supplementary material for: Antiepileptic drugs in glioblastoma survival: dichotomic or treatment and mechanism of action-dependent variable?
Source: Neurooncol Adv. 2026 Feb 11;8(1):vdag035. doi: 10.1093/noajnl/vdag035 (PMC12990308; doi:10.1093/noajnl/vdag035)
Supplement: vdag035_Supplementary_Data [file vdag035_supplementary_data.zip › Supplementary figures 1, 2, 4, 5.docx]

|  | **Restricted mean** | **Standard error** | **Median Survival** | **95% CI**  **Lower Upper** | |
| --- | --- | --- | --- | --- | --- |
| **All patients** |  |  |  |  |  |
| None | 13.7 | 2.35 | 9.01 | 7.40 | 13.1 |
| Radiotherapy | 18.0 | 7.78 | 8.03 | 6.03 | 17.7 |
| Chemotherapy | 24.1 | 8.73 | 14.40 | 13.2 | 20.0 |
| Chemoradiotherapy | 34.5 | 3.86 | 20.17 | 17.5 | 23.3 |
| **AED** |  |  |  |  |  |
| None | 11.6 | 2.43 | 7.53 | 5.13 | 12.6 |
| Radiotherapy | 10.5 | 1.64 | 8.03 | 5.90 | 17.8 |
| Chemotherapy | 17.8 | 3.99 | 14.9 | 13.6 | 26.1 |
| Chemoradiotherapy | 35.8 | 4.45 | 21.2 | 18.1 | 23.8 |
| **No AED** |  |  |  |  |  |
| None | 18.3 | 5.01 | 13.4 | 7.70 | 17.6 |
| Radiotherapy | 23.1 | 13.1 | 7.77 | 6.03 | - |
| Chemotherapy | 25.6 | 15.4 | 8.37 | 5.83 | - |
| Chemoradiotherapy | 25.8 | 4.65 | 17.3 | 12.2 | 25.7 |

|  | **Restricted mean** | **Standard error** | **Median Survival** | **95% CI**  **Lower Upper** | | **P value** |
| --- | --- | --- | --- | --- | --- | --- |
| **AED use overall** |  |  |  |  |  |  |
| AED | 23.8 | 2.3 | 16.2 | 13.7 | 18.1 |  |
| No AED | 27.0 | 5.4 | 13.8 | 10.9 | 17.5 | 0.78 |
| **6-month** |  |  |  |  |  |  |
| AED | 5.7 | 0.07 | * | - | - |  |
| No AED | 5.9 | 0.07 | * | - | - | 0.66 |
| **12-month** |  |  |  |  |  |  |
| AED | 10.2 | 0.23 | * | - | - |  |
| No AED | 10.1 | 0.36 | * | - | - | 0.32 |
| **18-month** |  |  |  |  |  |  |
| AED | 13.4 | 0.40 | 16.2 | 13.7 | - |  |
| No AED | 12.7 | 0.66 | 13.8 | 10.9 | 17.5 | 0.16 |
| **24-month** |  |  |  |  |  |  |
| AED | 15.5 | 0.56 | 16.2 | 13.7 | 18.1 |  |
| No AED | 14.2 | 0.90 | 13.8 | 10.9 | 17.5 | 0.27 |

**Supplementary Figure 1.**

**Supplementary Figure 2.**

|  |  | |  |  | **12 months** | **24 months** |  |
| --- | --- | --- | --- | --- | --- | --- | --- |
|  | |  | **Nº (%)** | **HR (95% CI, p value)** | **HR (95% CI, p value)** | **HR (95% CI, p value)** | |
| SV2 modulation | | Yes | 156 (66.1) | - | - | - | |
|  | | No | 90 (33.9) | 0.95 (0.72 – 1.25, p = 0.695) | 1.06 (0.68 – 1.64, p = 0.808) | 1.00 (0.73 – 1.36, p = 0.981) | |
| Increase GABA and voltage gated Na channel blocker | | No | 220 (93.2) | - | - | - | |
|  | | Yes | 16 (6.8) | 0.81 (0.48 – 1.36, p = 0.418) | 0.78 (0.32 – 1.92, p = 0.585) | 0.61(0.31 – 1.19, p = 0.144) | |
| Voltage gated Ca channel blocker | | No | 218 (92.4) | - | - | - | |
|  | | Yes | 18 (7.6) | 1.43 (0.88 – 2.33, p = 0.148) | 1.39 (0.67 – 2.88, p = 0.374) | 1.23 (0.71 – 2.13, p = 0.454) | |
| Voltage gated Na channel blocker | | No | 188 (79.7) | - | - | - | |
|  | | Yes | 48 (20.3) | Initial HR with time-dependent variable: 2.05 (0.80 – 5.28, p = 0.136) | 0.56 (0.31 – 1.03, p = 0.064) | **0.67 (0.45 – 0.99, p = 0.045)** | |

**Supplementary Figure 4.**

|  |  | |  |  | **12 months** | **24 months** |  |
| --- | --- | --- | --- | --- | --- | --- | --- |
|  | |  | **Nº (%)** | **HR (95% CI, p value)** | **HR (95% CI, p value)** | **HR (95% CI, p value)** | |
| SV2 modulation | | No | 80 (33.9) | - | - | - | |
|  | | Yes | 156 (66.1) | 1.25 (0.88 – 1.77  , p = 0.210) | 1.23 (0.75 – 1.23 p = 0.404) | 1.25 (0.88 – 1.77  , p = 0.210) | |
| Increase GABA and voltage gated Na channel blocker | | No | 220 (93.2) | - | - | - | |
|  | | Yes | 16 (6.8) | 0.64 (0.29 – 1.38, p = 0.250) | 0.47 (0.16 – 1.42, p = 0.182) | 0.64 (0.29 – 1.38, p = 0.250) | |
| Voltage gated Ca channel blocker | | No | 218 (92.4) | - | - | - | |
|  | | Yes | 18 (7.6) | 1.37 (0.74 – 2.53, p = 0.351) | 1.33 (0.61 – 2.92, p = 0.473) | 1.37 (0.74 – 2.53, p = 0.351) | |
| Voltage gated Na channel blocker | | No | 188 (79.7) | - | - | - | |
|  | Yes | | 48 (20.3) | Initial HR with time-dependent variable:1.96 (0.71 – 5.42, p = 0.19) | 0.81 (0.43 – 1.53, p = 0.517) | 0.81 (0.52 – 1.27, p = 0.361) | |

**Supplementary Figure 5.**
