# Supplementary material for: Antiepileptic drugs in glioblastoma survival: dichotomic or treatment and mechanism of action-dependent variable?
Source: Neurooncol Adv. 2026 Feb 11;8(1):vdag035. doi: 10.1093/noajnl/vdag035 (PMC12990308; doi:10.1093/noajnl/vdag035)
Supplement: vdag035_Supplementary_Data [file vdag035_supplementary_data.zip › Supplementary Figure legends.docx]

**Supplementary Figure 1.** Kaplan-Meier summary table and Log-rank test for AED use.

**Supplementary Figure 2.** Kaplan-Meier summary table for different adjuvant treatments for all patients, taking and not taking AEDs.

**Supplementary Figure 3.** Kaplan-Meier curves comparing overall survival in months for AED vs no AED use for different adjuvant treatments.

**Supplementary Figure 4.** Cumulative landmark analysis using COX regression test summary table per mechanism of action of AEDs with non-adjusted results. Model used for initial HR: $h(t\mid X,Z)=h0(t)\cdot exp(\beta1X(t)+\beta2Z1+\beta3Z2+...+\beta nZn-1)$, where X is Voltage gated Na channel blocker and Z are the rest proportional variables. Model used for cumulative landmark analysis: $h(t)=h0(t)exp(\beta1X1+\beta2X2+\cdots+\beta kXk)$ where X are all proportional variables.

**Supplementary Figure 5.** Cumulative landmark analysis using multivariate COX regression test summary table per mechanism of action of AEDs corrected for covariates known to be clinically relevant: Age, Gender, Resection, pre-operative and post-operative performance status, Response Assessment in Neuro-Oncology (RANO), and Methylated-DNA-protein-cysteine methyltrasferase (MGMT) status. Model used for initial HR: $h(t\mid X,Z)=h0(t)\cdot exp(\beta1X(t)+\beta2Z1+\beta3Z2+...+\beta nZn-1)$, where X is Voltage gated Na channel blocker and Z are the rest proportional variables. Model used for cumulative landmark analysis: $h(t)=h0(t)exp(\beta1X1+\beta2X2+\cdots+\beta kXk)$ where X are all proportional variables.

**Supplementary Figure 6.** Forest plot of a multivariate COX regression test per adjuvant treatment corrected displaying the effects of covariates known to be clinically relevant: Age, Gender, Type of resection, pre-operative and post-operative performance status, Response Assessment in Neuro-Oncology (RANO), and Methylated-DNA-protein-cysteine methyltrasferase (MGMT) status. Model used: $h(t)=h0(t)exp(\beta1X1+\beta2X2+\cdots+\beta kXk)$.

**Supplementary Figure 7.** Kaplan-Meier curves comparing survival of glioblastoma patients taking AEDs vs not taking AEDs that underwent adjuvant treatment. **A** Kaplan-Meier curve for overall survival in months. Median survival was not significantly different between patients taking AEDs and patients not taking AEDs (18.10 months vs 14.43 months respectively, hazard ratio (HR) = 1.05 (95% CI: 0.68-1.62, p = 0.81). **B** Kaplan-Meier curve for 24-month survival. Patients taking AEDs shown in orange, no AEDs in blue. Number of patients at risk for both groups are shown below the Kaplan-Meier curve. At 24 months, survival was 32.6% in patients not taking AEDs and 27.5% in patients taking AEDs (p = 0.19).

**Supplementary Figure 8.** Kaplan-Meier curves comparing survival of glioblastoma patients taking AEDs for seizure treatment vs for prophylactic/non-seizure treatment. There was no significant impact on survival (p = 0.886), with a median survival of 13.88 and 17.18 months respectively.
