# Supplementary figures and images for: Antiepileptic drugs in glioblastoma survival: dichotomic or treatment and mechanism of action-dependent variable?
Source: Neurooncol Adv. 2026 Feb 11;8(1):vdag035. doi: 10.1093/noajnl/vdag035 (PMC12990308; doi:10.1093/noajnl/vdag035)

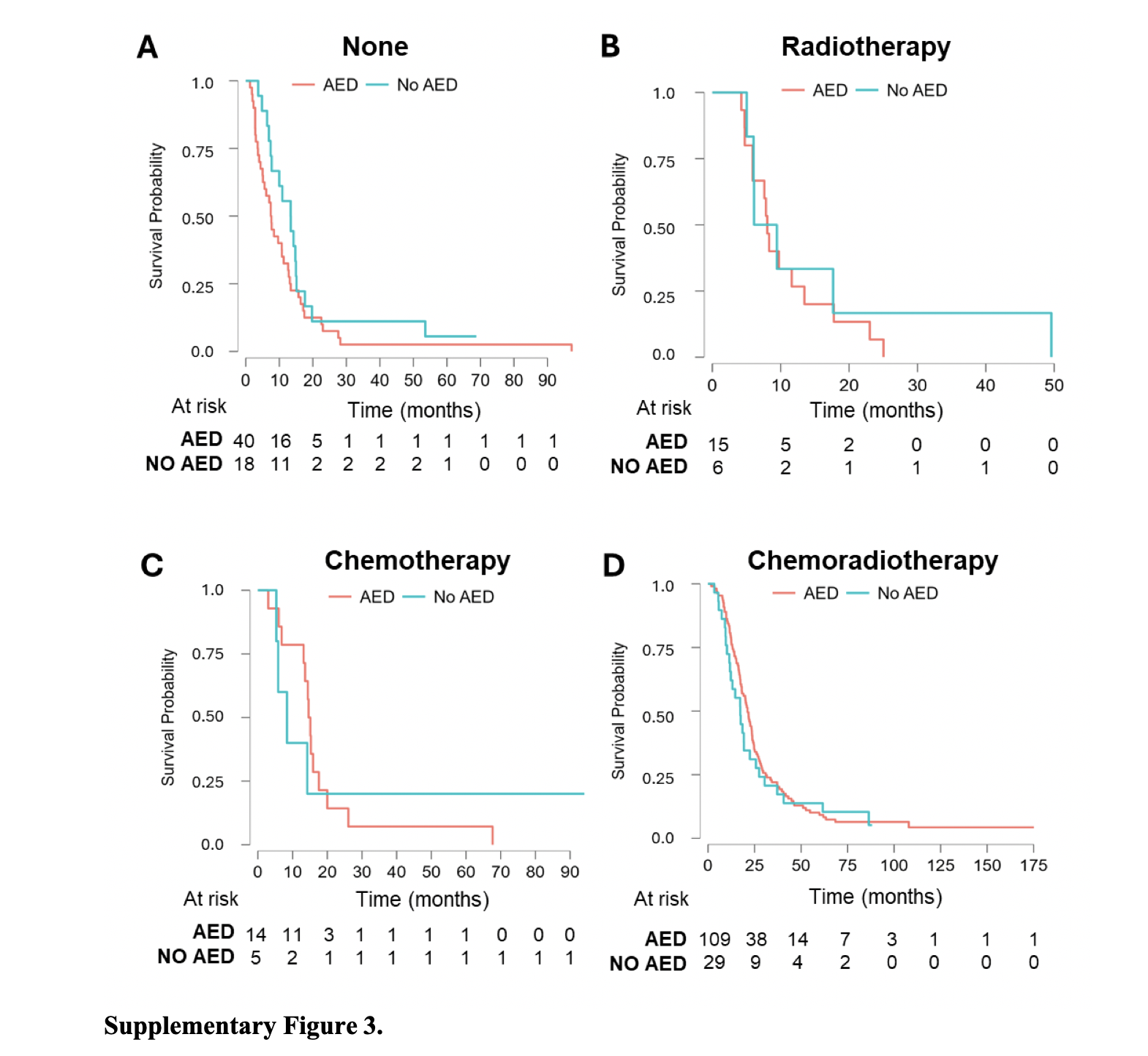

Supplement: vdag035_Supplementary_Data [file vdag035_supplementary_data.zip › Supplementary Figure 3.tiff]

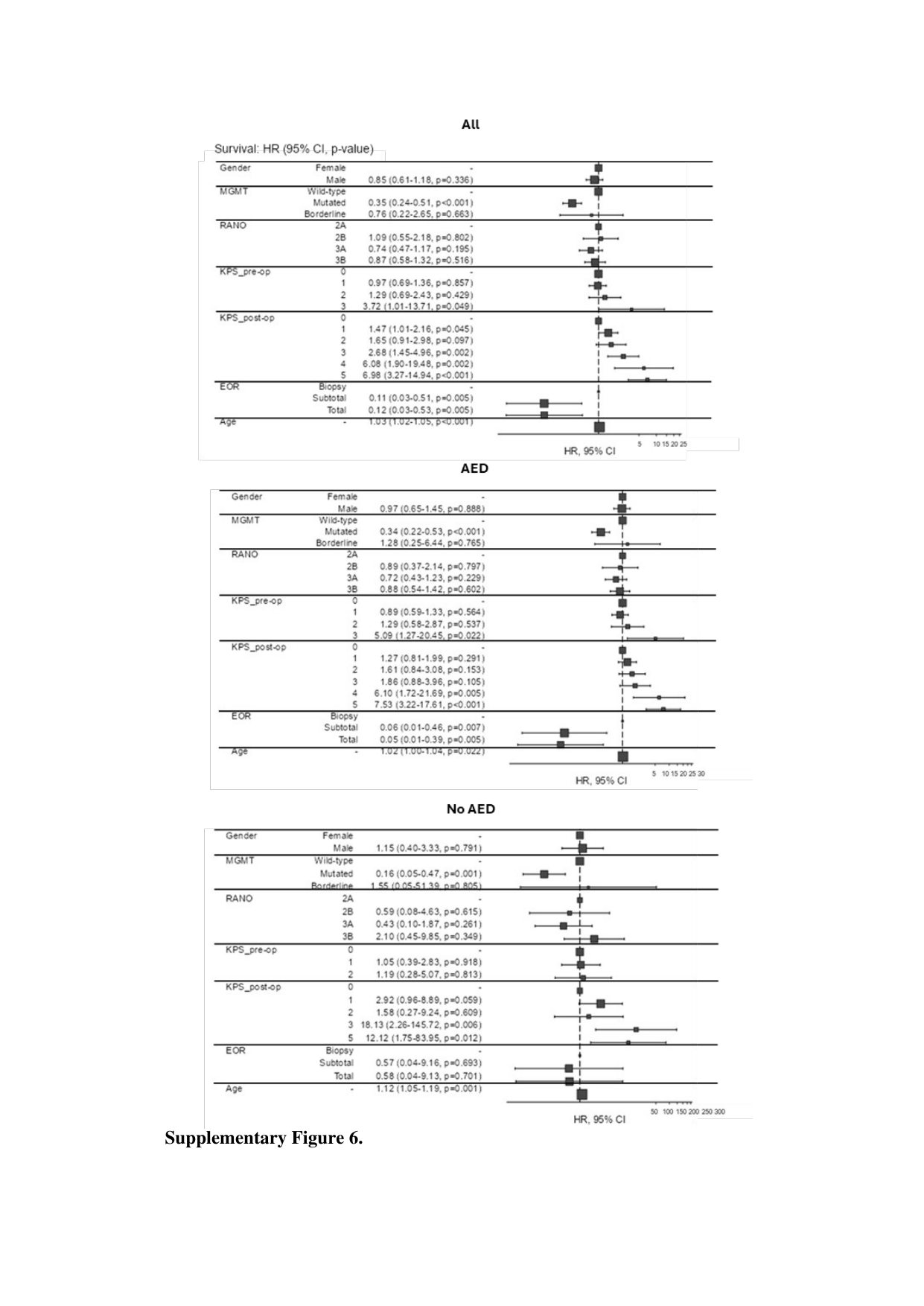

Supplement: vdag035_Supplementary_Data [file vdag035_supplementary_data.zip › Supplementary Figure 6.tiff]

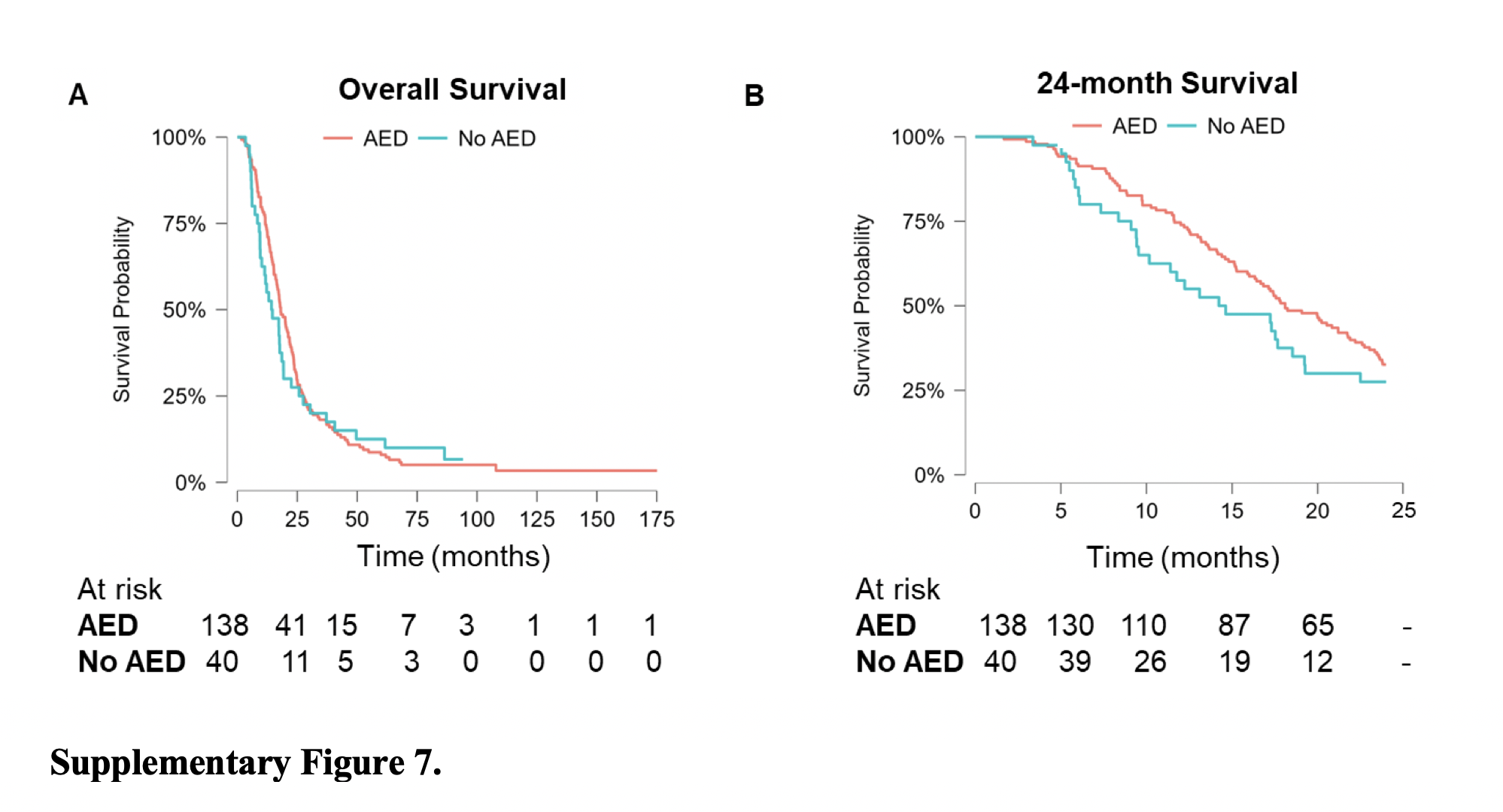

Supplement: vdag035_Supplementary_Data [file vdag035_supplementary_data.zip › Supplementary Figure 7.tiff]

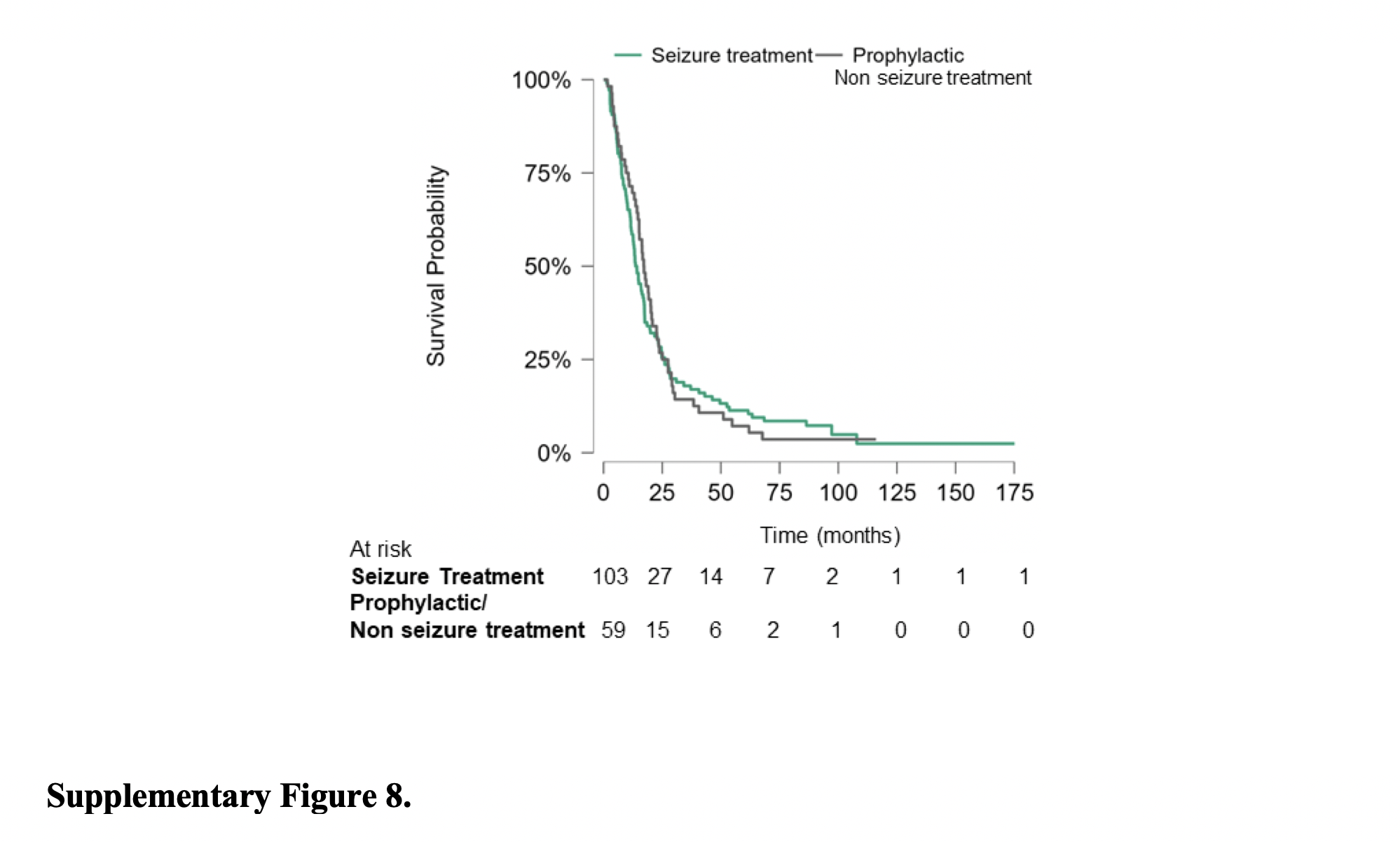

Supplement: vdag035_Supplementary_Data [file vdag035_supplementary_data.zip › Supplementary Figure 8.tiff]
